# Organic Acid Meat Decontamination: Optimising Application Parameters to Reduce the Microbial Load of *Yersinia enterocolitica* 4/O:3 on Pork

**DOI:** 10.17113/ftb.64.02.26.9340

**Published:** 2026-06-15

**Authors:** Marta Kiš, Jasenka Gajdoš Kljusurić, Nevijo Zdolec

**Affiliations:** 1 University of Zagreb, Faculty of Veterinary Medicine, Hygiene, Technology and Food Safety Unit, Heinzelova 55, Zagreb, Croatia; 2 University of Zagreb, Faculty of Food Technology and Biotechnology, Department of Process Engineering, Pierottijeva 6, Zagreb, Croatia

**Keywords:** *Yersinia enterocolitica* 4/O:3, pork, organic acids, water, decontamination

## Abstract

**Research background:**

Pigs are natural carriers of pathogenic bioserotypes of *Yersinia enterocolitica* and consumption of undercooked pork is a risk factor in the epidemiology of yersiniosis. The aim of this study is to determine the decontamination effect of lactic and acetic acids on pork cuts inoculated with *Yersinia enterocolitica* 4/O:3 strains (*N*=10) under laboratory conditions and to compare their effect with that obtained after washing with water.

**Experimental approach:**

A total of 20 decontamination protocols were carried out in which the effect of organic acid solutions (2 and 4 %) and water was investigated at different temperatures (25 and 80 °C) and exposure times (spraying for 10 and 30 s) at two time intervals (0 and 24 h).

**Results and conclusions:**

The decontamination effect obtained after application of lactic acid solutions was significantly higher (p<0.05) than that of acetic acid or water immediately after the treatment. After 24 h, their effects equalise (p>0.05), which can be attributed to the residual effect of the acids and the inadequate response of the cells to the cold conditions in the case of washing with water. Factor analysis showed that hot solutions applied for a longer exposure time had the greatest influence on the reduction of *Y. enterocolitica* 4/O:3 counts (p<0.05), in contrast to the type of acid and its volume fraction (p>0.05).

**Novelty and scientific contribution:**

This is the first organic acid susceptibility study focusing on the pathogenic bioserotype 4/O:3 of *Y. enterocolitica.* The study provides valuable insights for the development of strategies to control pathogenic *Y. enterocolitica* in the pork production chain and serves as a basis for future research.

## INTRODUCTION

The microbiological status of animal carcasses and portioned meat is a basic requirement for ensuring hygienically safe raw products. In relation to microbiological safety, the focus is primarily on pathogenic bacteria such as *Salmonella* spp., *Campylobacter* spp., *Yersinia enterocolitica* and *Escherichia coli*, whose presence is largely determined by the animal species and the meat. For example, *Salmonella* spp. and *Y. enterocolitica* are identified as priority biological hazards in the control of pork due to their occurrence in pigs, confirmed cases of human disease and epidemiological studies (*1*). Unlike *Salmonella* spp., most countries still do not have national control programmes for *Y. enterocolitica*, although the number of reported cases of yersiniosis is increasing (*2*).

Pigs are among the most common asymptomatic carriers of pathogenic strains of this bacterium, which mainly colonise the tonsils, lymph nodes and intestines (*3*-*5*). Serotypes O:3, O:8, O:9, and O:5,27 are most frequently associated with human infections (*6*, *7*), and according to European Food Safety Authority (EFSA) and European Centre for Disease Prevention and Control (ECDC) (*2*), the most common bioserotypes in Europe are 4/O:3 (86.9 %) and 2/O:9 (10.7 %). In many countries, bioserotype 4/O:3 remains by far the most frequently isolated from pigs (*8*-*11*), indicating the significant role of pigs in the epidemiology of human yersiniosis. The link between consumption of contaminated pork products and yersiniosis has been documented in several studies from different countries (*12*-*14*).

In this context, the processing of pig carcasses in the slaughterhouse is considered most important for preventing contamination of the meat and thus the entry of *Y. enterocolitica* into the pork production chain. Standard intervention methods, which have contributed to a lower prevalence of *Y. enterocolitica* on meat, mostly include procedures such as bagging the rectum, omitting the incision of lymph nodes during veterinary *post mortem* meat inspection, and separating the tonsils from the rest of the carcass. However, these measures are usually insufficient to completely eliminate the risk, as the pathogen is very common in the tonsils (*15*) and is psychrotrophic. This was confirmed by a recent study in Croatia (*16*), in which pathogenic *Y. enterocolitica* 4/O:3 was found in 101 of 234 (43.16 %) pig tonsil samples examined.

The use of organic acids in the decontamination of carcasses and meat cuts has been the subject of numerous studies for many years (*17*-*19*) and is considered as a way to improve the hygienic condition of the carcasses and thus the safety of the meat, taking into account the antimicrobial effect on pathogenic microorganisms (*20*). However, it is important to emphasise that decontamination methods must be part of an integrated food safety system and are not a substitute for good hygiene or manufacturing practices. Lactic acid and acetic acid are among the most studied organic acids to date (*18*, *21*, *22*), as they are widely distributed in nature and also used in the food industry (*23*). Regarding other pathogenic bacteria in meat hygiene, the literature review revealed a lack of data on the decontamination effect of organic acids against the *Y. enterocolitica* population on pork (*24*) and on the comparison of their efficacy with conventional washing methods using potable water.

Potable water is currently the only decontamination agent authorised in the EU for slaughtered carcasses, at least for pigs. The use of all other potential substances for decontamination purposes requires prior authorisation by the European Commission. Considering the EFSA Scientific Opinion (*25*), which reviews the decontamination potential of organic acids in reducing microbiological surface contamination of pig carcasses using the example of cattle carcasses (*26*), the aim of this study is to evaluate the decontamination effect of lactic and acetic acid on pork inoculated with different strains of *Y. enterocolitica* 4/O:3 and to compare their effect with that obtained after washing with water under the same conditions. The use of different volume fractions and temperatures of the solutions, as well as the duration of the decontamination protocol, will provide information on the optimal protocol that leads to the most desirable reductions in bacterial counts. Due to the complexity of the research methodology required on the slaughter line, the entire study was conducted under laboratory conditions.

## MATERIALS AND METHODS

### Experiment plan

Based on the selected factors (type of organic acid, acid volume fraction, solution temperature and exposure time), a factorial experimental design was established to determine the minimum number of protocols per *Yersinia enterocolitica* 4/O:3 strain. Considering four factors with two levels each, a 2×2×2×2 design was used, resulting in a total of 16 protocols. An additional four protocols were included using water at two temperatures and two exposure times, bringing the total number of protocols to 20 (Table 1). The reduction in the number of *Y. enterocolitica* 4/O:3 was verified at two time points (0 and 24 h) in triplicate.

**Table 1:** List of decontamination protocols used in the experiment

	Decontamination solution		
Protocol No.	*φ*(acetic acid)/%	Temperature/°C	*t*/s
1.	2	25	10
2.	4	25	10
3.	2	25	30
4.	4	25	30
5.	2	80	10
6.	4	80	10
7.	2	80	30
8.	4	80	30
	*φ*(lactic acid)/%		
9.	2	25	10
10.	4	25	10
11.	2	25	30
12.	4	25	30
13.	2	80	10
14.	4	80	10
15.	2	80	30
16.	4	80	30
	Water		
17.		25	10
18.		25	30
19.		80	10
20.		80	30

### Preparation of Y. enterocolitica 4/O:3 strains

*Y. enterocolitica* 4/O:3 strains (*N*=10) used in the experiment were collected as part of a previous study (*16*) and stored in a microbank cryopreservation system (Deltalab, Barcelona, Spain) at -80 °C until testing. Immediately before inoculation onto meat, the strains were cultured in PSB broth (peptone, sorbitol and bile salts; Sigma-Aldrich, Merck, St. Louis, MO, USA), then serially diluted to obtain optimal dilutions for meat contamination. Each strain was treated separately with 20 different protocols.

### Preparation of organic acid solutions

The decontamination solutions were prepared by diluting concentrated (90–99.5 %) lactic and acetic acid (Merck, Darmstadt, Germany) with distilled water (Merck, Darmstadt). For the preparation of 2 and 4 % solutions, the required volumes of acids and water were calculated according to the following formula:


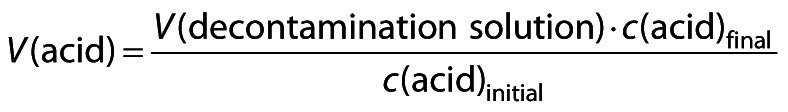
 /1/

The pH of the final solutions was measured prior to decontamination (pH meter pH50 VioLab; XS Instruments, Carpi, Italy).

### Meat decontamination using organic acid solutions

Decontamination was performed on fresh retail pork (*M. longissimus dorsi*), without skin or fat. For each protocol, a piece of meat with an average mass of 100 g, of the same size and thickness, was used, and the pH was measured before inoculation with *Y. enterocolitica* 4/O:3 strains. The meat was tempered to room temperature before inoculation. PSB broth containing *Y. enterocolitica* 4/O:3 strains was serially diluted 1:9 after 24 h of incubation at 30 °C, and 1 mL of the appropriate dilution was inoculated evenly over the meat surface with a sterile wand, achieving an average contamination of 4 log CFU/g (control). To simulate the contamination conditions in the slaughterhouse, the decontamination protocols were started 5 min after bacterial inoculation on the meat (cell adhesion). Water and prepared 2 and 4 % solutions of organic acids were applied at different temperatures (25 and 80 °C) and exposure times (10 and 30 s) to previously contaminated meat by spraying with an automatic sprayer (Gloria Haus und Garten, Gütersloh, Germany) at a pressure of 0.3 MPa from a distance of 10 cm. Five minutes after inoculation and before the start of decontamination, the meat was cut into two equal parts, one half serving as a positive control and the other half hung on a suitable rack for decontamination. Using a gravimetric diluter (Dilu*Flow*; Interscience, Cantal, France), the samples were weighed into a sterile bag, to which saline solution was added at a ratio of 1:10. After homogenisation for 1 min (BagMixer; Interscience), 0.1 mL was superficially inoculated onto CIN selective agar (Cefsulodin, Irgasan^TM^, Novobiocin; Sigma-Aldrich, Merck) according to the reference method. After incubation for 24 h at 30 °C, the number of colonies was determined using an automatic colony counter (Scan 1200; Interscience) and the result was expressed as log CFU/g. The same procedure was carried out on refrigerated samples after 24 h. The logarithmic values of the *Y. enterocolitica* 4/O:3 count after each decontamination protocol were subtracted from the values of the positive controls and are presented as the reduction in the *Y. enterocolitica* 4/O:3 count per gram of meat (R; log_10_). In addition, to exclude the possible presence of *Y. enterocolitica* in the meat due to contamination during production, a microbiological examination was performed on each individual meat package using the method described above. To determine the changes in the meat surface pH after decontamination, the pH of the solvent of the control and test samples was measured after homogenisation of each individual sample. All measurements were performed in triplicate and the results were expressed as mean value±S.D.

### Statistical analysis

The results were processed using descriptive statistical methods (Statistica, v. 13.5.0) (*27*) and presented as mean values of three measurements with standard deviation (x±S.D.). To determine statistically significant differences between the quantitative data, Student's *t*-test was used for indicators that followed a normal distribution and the Mann-Whitney *U* test was used for indicators that did not follow a normal distribution. The normality of the distribution was checked using the Kolmogorov-Smirnov test, with p=0.05. The correlation between the decrease in meat pH and bacterial reduction was tested using the correlation coefficient (r). Multivariate analysis tools were used for qualitative data processing. Factorial analysis of variance (factorial ANOVA) was used to examine the influence of the independent variables (factors) on the dependent variable (reduction in the number of *Y. enterocolitica* 4/O:3). Statistical significance was determined at the 0.05 level.

## RESULTS AND DISSCUSION

Decontamination with acidic solutions reduced the inoculated population of *Yersinia enterocolitica* 4/O:3 in all combinations of acid volume fraction, solution temperature, and decontamination duration. Their effect mostly depends on application temperature and exposure duration (p<0.05), in contrast to the acid type and volume fraction (p>0.05). Although lactic acid solutions produced the strongest initial decontamination effect, their impact after 24 h was equal to that of acetic acid solution and water.

Previous research on chemical decontamination of pork has mostly focused on lactic acid solutions (*28*-*31*) or combinations with acetic acid (*32*, *33*). Similar studies typically aim to reduce the overall microbiological contamination in terms of the total population of bacteria or enterobacteria, while studies on pathogenic bacteria focus on *Salmonella* spp., which makes comparison with the results for *Y. enterocolitica* somewhat difficult. Although some authors (*34*, *35*) point out that *Y. enterocolitica* is generally sensitive to organic acids, our results of the main reductions (Table 2) indicate that *Y. enterocolitica* 4/O:3 strain is more resistant to organic acids compared to the results of other studies (*28*, *32*, *36*). However, when comparing such results, all experimental parameters that may have influenced the reduction in bacterial load must be considered. These are primarily the type of acid, its volume fraction, temperature and method of application, duration of exposure, initial number of inoculated bacterial cells as well as the combinations of these variables, so a comparison of these results requires a critical approach (*37*).

**Table 2:** Comparison of the effectiveness of decontamination protocols based on the used factors (0 h)

	Acid	R*	Concentration	R	Temperature	R	Time	R
Decontamination protocol/reduction	1	(0.3±0.2)^a^	1	0.3±0.2	1	0.3±0.2	1	0.3±0.2
	9	(0.7±0.2)^a^	2	0.4±0.2	5	0.5±0.12	3	0.5±0.2
	2	(0.4±0.2)^b^	3	0.5±0.2	2	0.4±0.2	2	0.4±0.2
	10	(0.7±0.1)^b^	4	0.5±0.2	6	0.5±0.2	4	0.5±0.2
	3	(0.5±0.2)^c^	5	0.5±0.2	3	(0.5±0.2)^a^	5	(0.5±0.2)^b^
	11	(0.8±0.3)^c^	6	0.5±0.2	7	(0.9±0.4)^a^	7	(0.9±0.4)^b^
	4	(0.5±0.2)^d^	7	0.9±0.4	4	(0.5±0.2)^b^	6	(0.5±0.2)^c^
	12	(0.9±0.3)^d^	8	0.9±0.4	8	(0.9±0.4)^b^	8	(0.9±0.4)^c^
	5	0.5±0.2	9	0.7±0.2	9	0.7±0.2	9	0.7±0.2
	13	0.7±0.3	10	0.7±0.1	13	0.7±0.3	11	0.8±0.3
	6	(0.5±0.2)^e^	11	0.8±0.3	10	0.7±0.1	10	0.7±0.1
	14	(0.8±0.3)^e^	12	0.9±0.3	14	0.8±0.3	12	0.9±0.3
	7	(0.9±0.4)^f^	13	0.7±0.3	11	(0.8±0.3)^c^	13	(0.7±0.2)^d^
	15	(1.2±0.2)^f^	14	0.8±0.3	15	(1.2±0.2)^c^	15	(1.2±0.2)^d^
	8	0.9±0.4	15	1.2±0.2	12	(0.9±0.3)^d^	14	(0.8±0.3)^e^
	16	1.2±0.4	16	1.2±0.4	16	(1.2±0.4)^d^	16	(1.2±0.4)^e^

*N(*Y. enterocolitica* 4/O:3)_reduction_/(log_10_ CFU/g), **Values marked with the same letter in superscript within the same column are statistically different at p=0.05

When comparing the differences between the decontamination protocols using different types of organic acids, lactic acid was found to be significantly more effective (p<0.05) in all cases except when using 2 % hot acid solutions for 10 s and 4 % hot acid solutions for 30 s (Table 2). In these protocols, the reduction was also higher when using lactic acid, but without statistical significance (p>0.05). This is consistent with the results of most previous studies on a similar topic (*23*, *38*-*41*), in which lactic acid had a better decontamination effect than acetic acid. Although both lactic and acetic acids in their undissociated forms have a stronger bactericidal or bacteriostatic effect than inorganic acids, there is a difference in antimicrobial properties between the two. This is defined as the specific effect of the acid, which is known to be most pronounced in lactic acid (*35*), supporting our results. However, the above-mentioned differences between the observed protocols were not significant even after 24 h (p>0.05), mainly due to the increase in reduction values observed after acetic acid treatment (Table 3).

**Table 3:** Comparison of the effectiveness of decontamination protocols based on the used factors (24 h)

	Acid	R*	*φ*(acid)/%	R	Temperature/°C	R	*t*/s	R
Decontamination protocol/reduction	1	0.7±0.3	1	0.7±0.3	1	0.7±0.3	1	0.7±0.3
	9	0.8±0.2	2	0.7±0.3	5	0.6±0.3	3	0.8±0.2
	2	0.7±0.3	3	0.8±0.2	2	0.7±0.3	2	0.7±0.3
	10	0.6±0.2	4	0.8±0.2	6	0.7±0.2	4	0.8±0.2
	3	0.8±0.2	5	0.6±0.3	3	0.8±0.2	5	(0.6±0.3)^e^
	11	0.8±0.2	6	0.7±0.2	7	1.0±0.5	7	(1.0±0.5)^e^
	4	0.8±0.2	7	1.0±0.5	4	(0.8±0.2)^b^	6	(0.7±0.2)^f^
	12	0.9±0.3	8	1.2±0.6	8	(1.2±0.6)^b^	8	(1.2±0.6)^f^
	5	0.6±0.3	9	0.8±0.2	9	0.8±0.2	9	0.8±0.2
	13	0.8±0.3	10	0.6±0.2	13	0.8±0.3	11	0.8±0.2
	6	(0.7±0.2)^a^	11	0.8±0.2	10	(0.6±0.2)^c^	10	(0.6±0.2)^g^
	14	(1.0±0.3)^a^	12	0.9±0.3	14	(1.0±0.3)^c^	12	(0.9±0.3)^g^
	7	1.0±0.5	13	0.8±0.3	11	(0.8±0.2)^d^	13	(0.8±0.3)^h^
	15	1.2±0.3	14	1.0±0.3	15	(1.2±0.3)^d^	15	(1.2±0.3)^h^
	8	1.2±0.6	15	1.2±0.3	12	0.9±0.3	14	1.0±0.3
	16	1.1±0.4	16	1.1±0.4	16	1.1±0.4	16	1.1±0.4

* *N*(*Y. enterocolitica* 4/O:3)_reduction_/(log_10_ CFU/g)

**Values marked with the same letter in superscript within the same column are statistically different at level 0.05

This can be attributed to the stronger residual effect of acetic acid on meat, which, despite its weaker effect immediately after the treatment, considerably impedes the growth and survival of the remaining live but damaged bacterial cells, especially when compared with untreated bacterial cells from control samples. Dan *et al.* (*32*) reached the same conclusions, finding that 3-5 % acetic acid solutions were more effective in reducing *Yersinia* spp. counts. In this respect, no significant differences were found in the case of lactic acid due to its initially better antimicrobial effect at 0 h, which supports the previously mentioned conclusions of most authors.

There was no statistically significant difference (p>0.05) in the reduction of pathogens when using different volume fractions of acetic or lactic acid solutions at the same exposure time and solution temperature. The same results were recorded after 24 h. Regarding the concentration of acids used, although numerous studies have shown that their increase correlates with a reduction in the bacterial count (*40*, *42*-*44*), in our case, the use of 4 % solutions of both types of acid did not lead to a statistically greater reduction in the *Y. enterocolitica* 4/O:3 population. However, some authors (*45*, *46*) have reached the same conclusion when using an even wider range of acid volume fractions. Considering the pH values of these solutions or the small variations between the volume fractions used, it can be expected that 4 % acid solutions are not sufficient to induce a stronger antimicrobial effect in most cases, at least not to the same extent as other observed parameters.

**Fig. 1:**
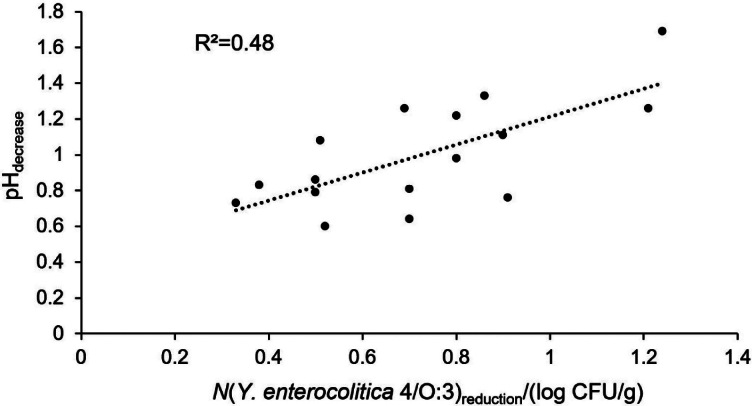
Correlation between the decrease in the meat pH value and the reduction in the number of *Y. enterocolitica* 4/O:3 (r=0.69)

Thus, comparing the decrease in the meat pH after decontamination with the reduction of *Y. enterocolitica* 4/O:3 populations, it was found that the decrease in pH correlated moderately with the degree of reduction (Fig. 1), *i.e.* a greater decrease in meat pH led to a greater reduction in the *Y. enterocolitica* 4/O:3 population (*r*=0.69, p<0.01). A linear regression model confirmed this relationship, showing that approx. 48 % of the variation in bacterial reduction can be explained by the pH decrease (R^2^=0.48). Thus, viewed individually, it was found that in more than 50 % of protocols, a statistically significant decrease in the pH of decontaminated meat did not lead to a statistically significant reduction in the inoculated population of *Y. enterocolitica* 4/O:3, supporting the insignificance of the acid solution volume fraction. This is best illustrated by aggregate pH values of meat measured after 24 h of cooling, where, under the influence of strong buffer capacity, the pH returns to physiological levels (data not shown), while a simultaneous decline in the population of inoculated bacterial cells is observed. The above supports the consideration of other potential negative consequences of decontamination, such as the effects on the organoleptic properties of meat, especially in the case of acetic acid, where even a small increase in volume fraction can negatively affect meat odour.

When temperature was considered as a factor, a significantly higher (p<0.05) reduction was achieved with hot acid solutions, but only during the longer exposure time of 30 s. Exposure for 30 s significantly (p<0.05) reduced the bacterial population compared to a shorter exposure of 10 s, but only when hot solutions were used. This was confirmed by a factorial analysis (Table 4), which showed that the use of acidic solutions at different temperatures with shorter or longer exposure times was a more significant factor (p<0.05) in reducing bacterial populations, with higher temperatures and longer decontamination protocols being mutually dependent.

**Table 4:** The influence of the main decontamination factors and their interactions on the reduction in the number of *Y. enterocolitica* 4/O:3

Factor	Factor interaction and their significance						
Acid	0.096	Volume fraction	0.529	Temperature	0.700	Time	0.085
		Temperature	0.096	Time	0.058		
		Time	0.171	Volume fraction	0.653		
Volume fraction	0.532	Temperature	0.227	Time	0.404		
		Time	0.796				
Temperature	0.001	Time	0.044				
Time	0.000						

Values less than 0.05 were considered statically significant

The literature contains contradictory information on these factors, which can be attributed to the use of different experimental methods, making their interpretation difficult. For example, Christiansen *et al.* (*28*) found a significantly greater reduction in the number of *Salmonella* spp. when a 2.5 % lactic acid solution was applied for 5 s longer, while in the study by Laury *et al.* (*47*), even a 15-second longer application of the same type and volume fraction of acid had no effect on the differences in their numbers. Such differences are to be expected, especially considering the other parameters that differed, such as the type of raw material treated (pork steaks *vs* chicken carcasses with skin), the method of treatment (acid spraying *vs* immersion in solution), the temperature of the solution used (55-80 °C *vs* room temperature) and the number of bacterial cells inoculated (10^7^ CFU/cm^2^ *vs* 10^6^ CFU/mL). The complexity of comparing such results is further illustrated in the aforementioned study by Christiansen *et al*. (*28*), in which a better decontamination effect was achieved through longer treatment and higher temperature of the applied solutions, despite some parameters favouring reduced sensitivity or a weaker inhibitory effect. For example, the volume fraction of the lactic acid solution was lower than ours, the exposure time was shorter, the adhesion time of the bacteria was significantly longer, and the count of bacteria per cm^2^ was higher, with the note that, in addition to *Y. enterocolitica*, *Salmonella* spp. and *E. coli* were also inoculated. The latter may explain the increased reduction due to the weak competitiveness of *Y. enterocolitica*. However, drawing any conclusions requires data with clearly equal parameters, which are far from sufficient in the case of *Yersinia*. Our results showed a slightly higher reduction in the number of *Y. enterocolitica* 4/O:3 in all protocols with prolonged exposure, but as we have already mentioned, only the reduction using hot acidic solutions was statistically significantly higher. A possible explanation for this lies in the temperature of the solution, which was heated to 80 °C immediately before application, but cooled down quickly during spraying and thus had lower efficiency, which was then compensated for by a longer application of 30 s. As there were no such significant differences when cold solutions were used, the observed effects are probably due to mechanical influences or the rinsing effect.

Guided by the recommendations of the EFSA Scientific Opinion (*25*), which points out the lack of scientific evidence comparing the decontamination effect of lactic acid and water as the only authorised decontaminant in pigs, another objective of this study was to determine whether the application of water under the same conditions leads to an equivalent reduction in the number of *Y. enterocolitica* 4/O:3 on meat. Washing (spraying) carcasses with water is a routine procedure on the slaughter line that has been shown to reduce the present microorganisms (*20*). The effectiveness of washing with water has been observed in research mostly in connection with the inclusion of water as an additional agent in decontamination protocols with organic acids, and usually such combinations led to the greatest reductions in the target bacterial population (*31*, *48*, *49*).

Comparisons of identical decontamination protocols (in terms of temperature and duration) with acetic acid, lactic acid and water are shown in Table 5. In contrast to acetic acid, protocols with lactic acid showed a significantly higher efficacy in reducing the inoculated population of *Y. enterocolitica* 4/O:3 on meat than standard protocols with water. A statistically higher reduction (p<0.05) at 0 h was observed in all protocols with lactic acid than in water, except in protocol no. 13. Surprisingly, compared to 0 h, after 24 h there were no significant differences in the reduction of *Y. enterocolitica* 4/O:3 count in decontaminated meat between the observed protocols (p>0.05).

**Table 5:** Comparison of the decontamination effect of acetic acid, lactic acid and water solutions against *Y. enterocolitica* 4/O:3 strains

Decontamination protocol				*N*(*Y. enterocolitica* 4/O:3)_reduction_/(log_10_ CFU/g)	
Decontamination solution				*t*(after decontamination)/h	
*φ*(acid)/%		Temperature/°C	*t*/s	0	24
acetic 2		25	10	0.3±0.2	0.7±0.3
lactic 2		25	10	(0.7±0.2)^a^	0.8±0.2
acetic 4		25	10	0.4±0.2	0.7±0.3
lactic 4		25	10	(0.7±0.1)^b^	0.6±0.2
0	water	25	10	(0.4±0.1)^ab^	0.6±0.2
acetic 2		25	30	0.5±0.2	0.8±0.2
lactic 2		25	30	(0.8±0.3)^c^	0.8±0.2
acetic 4		25	30	0.5±0.2	0.8±0.2
lactic 4		25	30	(0.9±0.3)^d^	0.9±0.3
0	water	25	30	(0.6±0.1)^cd^	0.8±0.2
acetic 2		80	10	0.5±0.2	(0.6±0.3)^h^
lactic 2		80	10	0.7±0.3	0.8±0.3
acetic 4		80	10	0.5±0.2	(0.7±0.2)^i^
lactic 4		80	10	(0.8±0.3)^e^	1.0±0.3
0	water	80	10	(0.5±0.2)^e^	(0.9±0.3)^hi^
acetic 2		80	30	0.9±0.4	1.0±0.5
lactic 2		80	30	(1.2±0.2)^f^	1.2±0.3
acetic 4		80	30	0.9±0.4	1.2±0.6
lactic 4		80	30	(1.2±0.4)^g^	1.1±0.4
0	water	80	30	(0.8±0.1)^fg^	0.9±0.1

Results are presented as mean value±S.D, *N*=3. Values marked with the same lowercase letters in superscript in the same column differ statistically at p=0.05. Differences between the individual protocols with acetic acid and lactic acid are shown in the previous tables

**Fig. 2:**
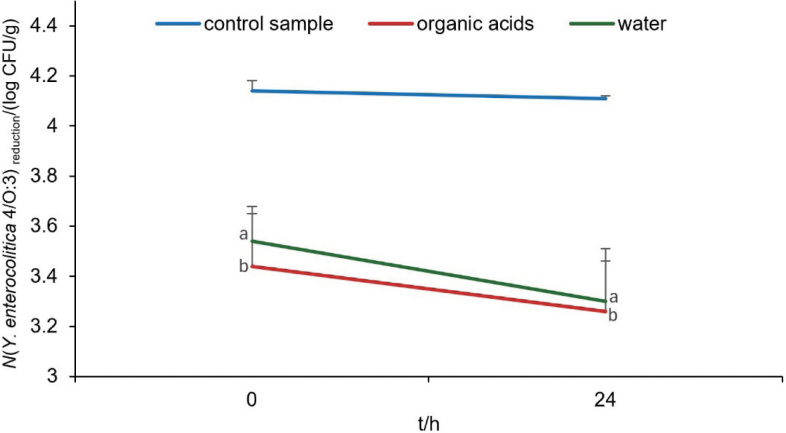
*Y. enterocolitica* 4/O:3 count after decontamination protocols with organic acid solutions and water at 0 and 24 h. Results are presented as mean value±S.D. Values marked with the same lowercase letters are statistically different at the 0.05 level

The comparison of the two measurement intervals (0 and 24 h) within the test and control samples clearly shows that the use of decontamination protocols with organic acid solutions, in addition to the initial reduction in the number of *Y. enterocolitica* 4/O:3, also results in a further reduction of the population during meat chilling (Fig. 2). This was also observed in the control samples, but to a lesser extent and without statistical significance. This is not surprising considering the psychrotrophic nature of *Y. enterocolitica*, which allows it to survive under chilling conditions, so that its numbers are maintained within a stable population with only minor variations. In the treated samples, immediately after exposure, the inoculated bacterial population decreases and the number of surviving but damaged bacterial cells stagnates and decreases over time. The reason for this is the aforementioned residual effect of organic acids, which, according to Rodriguez-Melcon *et al.* (*46*), persists in the case of lactic acid even 120 h after application and thus leads to an additional reduction in the bacterial population (>2 log) compared to the control sample. This explains the greater reductions achieved in our study, which, despite the initially stronger effect of lactic acid, led to an equalisation of its effect with that of acetic acid (Table 6).

**Table 6:** Average reduction of *Y. enterocolitica* 4/O:3 counts after decontamination protocols with organic acids and water

	*N*(*Y. enterocolitica* 4/O:3)_reduction_/(log CFU/g)	
Solution	*t*(after decontamination)/h	
	0	24
Acetic acid	(0.6±0.2)^Aa^	(0.8±0.2)^a^
Lactic acid	(0.9±0.2)^AB^	(0.9±0.2)
Water	(0.6±0.2)^Bb^	(0.8±0.2)^b^

Results are presented as mean value±S.D. Values marked with the same capital letter in superscript in the same column are statistically different at the 0.05 level. Values marked with the same lowercase letters in superscrript in the same row are statistically different at the 0.05 level

As for water, given that in this case there was no residual influence of acids or low pH, it is difficult to determine with certainty the reason for the above results. Since the differences between the control and test samples indicate a different development in the number of bacterial cells, it is understandable to consider the role of water application in this context. After the application of water, the meat is rapidly cooled to 4 °C, with the water remaining on the surface contributing to accelerated cooling and exposure of the bacterial cells to the so-called ’cold reaction’. As numerous authors have noted (*50*-*52*), the ability to adapt to such temperature differences must be accompanied by a series of complex biochemical and cellular changes in the bacterial cell, such as membrane permeability, protein conformation or gene expression. In the study by Li *et al.* (*53*), the physiological processes of the cold response in *Y. enterocolitica* were investigated by comparing growth ability, gene and protein expression, and cell motility after exposure to cold conditions. The cold response was induced in 55 strains of *Y. enterocolitica* of different bioserotypes, and the results showed, among other things, that the degree of induction of such a response is strain-specific and temporally conditioned by adaptation to cold conditions. Relating the above statements to our studies, it is possible that the decrease in the number of *Y. enterocolitica* 4/O:3 after 24 h of refrigeration is due to their low adaptation to the cold conditions, especially since such a tendency was not present in the untreated control samples.

The relatively low reduction of *Y. enterocolitica* 4/O:3 count achieved in this type of research, *i.e.* under laboratory conditions, is not necessarily to be expected when it is carried out under industrial conditions, *i.e.* on the slaughter line. Indeed, the conditions on the slaughter line differ considerably from those in the laboratory, starting with the meat as a treatment medium, the microclimatic conditions to which the meat is exposed, and the decontamination process itself. The number of bacterial cells of 10^3^ or 10^4^ per gramme or cm^2^ used in such studies are unlikely to occur at the slaughter line, severely obstructing the survival of *Yersinia*, which is otherwise not very competitive, especially compared to other psychrotrophic bacteria. It is assumed that the contamination of carcasses with *Y. enterocolitica* at the slaughter line is less than 1 log CFU/cm^2^ (*54*, *55*), apart from the fact that these cells are not adapted to acidic conditions. In this respect, the bacterial cells used in laboratory tests are generally in a stationary growth phase during inoculation, which enables them to adhere better and thus react less sensitively to acid than would be the case with contamination under natural conditions (*56*). Furthermore, decontamination at the slaughter line is usually carried out on carcasses that are still warm, which shows a better decontamination effect than with chilled meat (*35*). The overgrowth of muscle with fatty tissue (which was not present in the laboratory meat sample) contributes to a better decontamination effect due to a weaker buffering capacity than in meat (*57*). In addition, the refrigeration conditions to which meat is exposed in the laboratory differ considerably from those in industry, where strong air currents cause a sudden drop in water activity, *i.e.* surface drying, which contributes to the reduction of surviving but damaged cells. Finally, under industrial conditions, the decontamination parameters themselves would be met by the application of a higher spray pressure in combination with an additional rinse with water.

However, when considering decontamination of the entire carcass and to predict the above-mentioned effects, it is necessary to conduct the same study on a skin model, which is more exposed to contamination on the slaughter line.

## CONCLUSIONS

Considering all the results of this work, the decontamination potential of both lactic and acetic acids in reducing the number of *Yersinia enterocolitica* 4/O:3 on pork is evident. The effect depends on the type of decontamination protocol, particularly the temperature of the solution and the duration of exposure. Regarding the type of acid, a better decontamination effect was obtained with lactic acid, and the reduction was greater when hot solutions and longer exposure time were used. However, after 24 h, the effect equalises with that observed after using acetic acid solution and water. This is the result of the growth stagnation of the untreated population of bacterial cells and the simultaneous decrease in the number of exposed cells. In the first case, we attribute this to the residual effect of the acids, and in the second case to the inadequate response of the cells to the cold conditions to which they are exposed immediately after treatment. Overall, our study provides valuable insights into the development of strategies to control pathogenic *Y. enterocolitica* in the pork production chain and serves as a basis for future research. Further studies are needed to evaluate the long-term effects of these methods on bacterial survival, the development of their resistance to acids, or the induction of a non-culturable state.
